# *De Novo* Assembly and Comparative Transcriptome Analysis Provide Insight into Lysine Biosynthesis in* Toona sinensis* Roem

**DOI:** 10.1155/2016/6735209

**Published:** 2016-06-07

**Authors:** Xia Zhang, Zhenqiao Song, Tian Liu, Linlin Guo, Xingfeng Li

**Affiliations:** ^1^State Key Laboratory of Crop Biology, Shandong Agricultural University, Tai'an, Shandong 271018, China; ^2^Agronomy College, Shandong Agricultural University, Tai'an, Shandong 271018, China

## Abstract

*Toona sinensis* Roem is a popular leafy vegetable in Chinese cuisine and is also used as a traditional Chinese medicine. In this study, leaf samples were collected from the same plant on two development stages and then used for high-throughput Illumina RNA-sequencing (RNA-Seq). 125,884 transcripts and 54,628 unigenes were obtained through* de novo* assembly. A total of 25,570 could be annotated with known biological functions, which indicated that the* T. sinensis* leaves and shoots were undergoing multiple developmental processes especially for active metabolic processes. Analysis of differentially expressed unigenes between the two libraries showed that the lysine biosynthesis was an enriched KEGG pathway, and candidate genes involved in the lysine biosynthesis pathway in* T. sinensis* leaves and shoots were identified. Our results provide a primary analysis of the gene expression files of* T. sinensis* leaf and shoot on different development stages and afford a valuable resource for genetic and genomic research on plant lysine biosynthesis.

## 1. Introduction

*Toona sinensis* (A. Juss.) Roem (also known as* Cedrela sinensis*, belongs to the family Meliaceae), a perennial hardwood referred to as Xiangchun in Chinese and Chinese Mahogany in English, is a native plant found in Asia. Its edible young leaves are consumed as a nutritious vegetable known as Xiangchun Ya in China due to their unique aromatic flavor and abundance of vitamins, minerals, and antioxidants [1, 2]. The leaves of* T. sinensis* are also employed in Chinese traditional medicine to treat diarrhea, chronic dysentery, bloody stool, seminal emissions, leucorrhoea, and metrorrhagia [1]. More recently, various other biological properties discovered in the* T. sinensis* leaf extracts have been reported, including antioxidant [3], anticancer [4], anti-inflammatory, antidiabetic [5], and antineoplastic [6] effects, as well as antiatherosclerotic potential [7], inhibitory effects against severe acute respiratory syndrome (SARS) coronavirus replication, antitumor effects [8], and even inhibitory effects against the pandemic influenza A (H1N1) virus [9].

As spring seasonal vegetables, the tender leaves and shoots of* T. sinensis* are fresh, flavorful, and delicious before “Guyu,” a day marking one of the 24 divisions of the solar year in the traditional Chinese calendar, which generally occurs on April 20th or 21st every year. After “Guyu,” the flavor of this vegetable decreases considerably, and the tender stems of* T. sinensis* become fibrous, thus affecting the taste.

*T. sinensis* leaves collected in early spring are rich in nutrients, including protein (average 3.06 mg/g fresh weight), soluble sugars (3.2 mg/100 g), fats (7.15 mg/100 g), and several essential amino acids (1.92 mg/g) [10, 11], such as lysine, valine, leucine, and isoleucine, with lysine found at its highest levels among leafy vegetables. Lysine is one of the most limited essential amino acids in vegetative foods consumed by humans and livestock. In addition to serving as a necessary building block for all proteins, lysine also plays a major role in regulating plant growth and responses to the environment as an important signaling amino acid [12]. However, advances in the understanding of metabolic regulation still suffer from insufficient research concerning the molecular basis of lysine synthesis in plants [13], particularly regarding research on transcriptional regulation, which is still limited [14].

*T. sinensis* has a long history of cultivation in China and is widely distributed throughout Asia [15]. Most of the previous studies on this species have focused on its cultivation, effective ingredients [2, 7], and pharmacological value [4]. Very limited information on the genetic basis of* T. sinensis* is available [16, 17], and there have been only a few reports addressing its genetic diversity using random amplified polymorphic DNA (RAPD) and isoenzyme analyses [16] and by examining terpene synthase genes [17]. Transcriptome researches would clearly improve understanding of the phylogeny and diversification of* T. sinensis* [18].

Recently, RNA-sequencing (RNA-Seq) has emerged as a powerful approach to performing transcriptome analysis directly through sequencing, thereby avoiding the need for prior knowledge of the transcriptome under consideration. As the cost of sequencing decreases, RNA-Seq is becoming more affordable for use in transcriptome studies, showing both high-throughput and high-resolution capabilities. Several previous transcriptome studies in various plant species using leaves or shoots as material have provided important information for understanding multiple aspects of the biochemistry, development, and metabolism of leaves and shoots [19–22], as well as novel insights into the biosynthesis of metabolic compounds [23, 24]. However, there are currently no published transcriptome studies on* T. sinensis*.

In this study, the transcriptomes of* T. sinensis* leaves and shoots collected at various times were sequenced using Illumina paired-end sequencing technology. The sequencing data were then assembled and annotated; candidate genes involved in the lysine biosynthesis pathway in* T. sinensis* leaves and shoots were identified. To the best of our knowledge, this is the first systematic report addressing the transcriptomes of* T. sinensis* leaves and shoots. The transcriptome data generated from this study provide crucial resources for gene annotation and discovery, molecular marker development, and genomic and transcriptomic assembly. Moreover, our results enhance our understanding of lysine biosynthesis in plants, which may prove valuable for future production of high-lysine crops.

## 2. Materials and Methods

### 2.1. Materials

*T. sinensis* tree used for RNA-Seq belongs to red young leaf, one of four main genotypes in China. The one used in this preparation had grown for 10 years in the Botanical Garden at Shandong Agricultural University. Fresh young shoots and leaves were collected on April 15th (abbreviated as XC-*L*-4) and old leaves and shoots on June 15th (abbreviated as XC-*L*-6) for use in this study. These specimens were obtained from the same tree. After harvesting, these samples were immediately frozen in liquid nitrogen and stored at −80°C until further processing.

### 2.2. RNA Isolation and Quality Verification

Total RNA was isolated from a mixture of equal amounts of leaves and shoots. To eliminate variation, three biological repetitions were performed for total RNA isolation. Equal amounts of high-quality RNA from the three biological repetitions were mixed for cDNA synthesis. Total RNA was extracted using TRIzol® reagent (Invitrogen, San Diego, USA) and then treated with DNase I (Invitrogen). RNA purity was checked using a NanoPhotometer® spectrophotometer (Implen, CA, USA). The RNA concentration was measured with the Qubit® RNA Assay Kit on a Qubit 2.0 Fluorometer (Life Technologies, CA, USA).

### 2.3. cDNA Library Construction and Sequencing

A 30 *μ*g mixed RNA sample, confirmed to show the RNA integrity number (RIN) value above 8.0, was used as input material to construct the sequencing library. The library was generated using the Illumina TruSeq*™* RNA Sample Preparation Kit (Illumina, San Diego, USA) following the manufacturer's recommendations. Clustering of the index-coded samples was performed on the cBot Cluster Generation System using the TruSeq PE Cluster Kit v3-cBot-HS (Illumina) according to the manufacturer's instructions. After cluster generation, the library preparations were sequenced on the Illumina HiSeq 2000 platform, and paired-end reads with a length of 100 bp were generated.

### 2.4. Data Processing, Assembly and Annotation

Clean data (clean reads) were obtained after trimming the adapter and low quality reads and removing those shorter than 50 bp from the raw data. At the same time, Q20 and Q30 values and the levels of GC content and sequence duplication were calculated for the clean data. All downstream analyses were based on clean data demonstrated to be high quality. The left files (read1 files) from all libraries/samples were pooled into one large left.fq file and the right files (read2 files) into one large right.fq file. Transcriptome assembly was accomplished based on the left.fq and right.fq files using Trinity [25].

All of the assembled unigenes of* T. sinensis* were subjected to searches against the Nr (NCBI nonredundant protein sequences) database to identify putative mRNA functions using the BLAST algorithm [26, 27] with an* E*-value cut-off of 10^−5^. Additionally, GO (gene ontology) terms were extracted from the best hits obtained from BLASTx searches against the Nr (nonredundant protein database) and PFAM (protein family) using the Blast2GO. The BLAST algorithm was also employed to align unique sequences to the following databases to predict possible functional classifications and molecular pathways: Nt (NCBI nonredundant nucleotide sequences), Swiss-Prot (a manually annotated and reviewed protein sequence database), KEGG (Kyoto Encyclopedia of Genes and Genomes), KO (KEGG Ortholog database), and KOG (euKaryotic Ortholog Groups) (with* E*-value cut-off of 10^−5^).

### 2.5. Gene Expression Pattern Analysis

Gene expression levels were estimated by mapping clean reads to the Trinity transcript assembly using RSEM [28] for each sample. The abundance of all genes was normalized and calculated using the uniquely mapped reads via the RPKM (reads per kilo bases per million reads) method [29]. Differential expression analysis of the two samples was performed by modeling count data with negative binomial distributions as described under the DEGseq method [30]. *P* values were adjusted using the *q* value [31], and a *q* value < 0.005 and |log⁡2  (fold change)|>1 were set as the thresholds for significantly differential expression. The identified differentially expressed genes (DEGs) were subjected to GO and KO enrichment analyses. GO enrichment analyses were performed using GOseq [32] based on the Wallenius noncentral hypergeometric distribution to map all DEGs to terms in the GO database (*P* value ≤ 0.05), searching for significantly enriched GO terms among the DEGs. KEGG pathway enrichment analysis of the DEGs was performed using KOBAS [33].

## 3. Results

### 3.1. RNA-Sequencing and* De Novo* Assembly

An overview of the sequencing results was shown in Table S1 in Supplementary Material available online at http://dx.doi.org/10.1155/2016/6735209. After cleaning and checking read quality, 4.26 Gb and 5.94 Gb of clean data were obtained, for sample (XC-*L*-4) and sample (XC-*L*-6), respectively. Among the clean reads, more than 90% exhibited a read quality of Q30 (sequencing error rate, 0.1%) or higher. The GC contents were approximately 45.09% and 43.45%, respectively. These reads were considered to be high-quality data for further analysis. Using the Trinity program, the short-read sequences were assembled into 125,884 transcripts with a mean length of 1,290 bp and an N50 value of 2,133 bp (Table 1). These transcripts were further clustered, resulting in 54,628 unigenes, with an N50 value of 1,304 bp and a mean length of 764 bp, among which 7,138 genes with length ranged from 1 kb to 2 kb and 5,158 genes with length greater than 1 kb, respectively.

### 3.2. BLASTx Similarity Analysis

All of the unigenes obtained from* T. sinensis* were subjected to the BLASTx similarity analysis against the Nr NCBI database, and 23,227 (42.51%) exhibited significant matches. Among these unigenes matched, 86.11% of the* T. sinensis* unigenes exhibited matches with 5 plant species, which were* Vitis vinifera* (30.75%), followed by* Populus trichocarpa* (24.15%),* Ricinus communis* (23.87%),* Glycine max* (5.26%), and* Arabidopsis thaliana* (2.08%), and the remaining of the unigenes (13.89%) matched more than 20 other species, each of which accounted for less than 2% of the hits.

In addition to using the NCBI Nr database, BLASTx searches for* T. sinensis* unigenes were conducted against Nt, PFAM, Swiss-Prot, KOG, GO, and KO databases. Among the 54,628 unigenes, 14,394 (26.34%) showed significant matches in the Nt database, 18,787 (34.38%) displayed significant matches in the GO database, 16,303 (19.84%) showed significant matches in the PFAM, and 16,627 (30.43%) exhibited similarity to proteins in the Swiss-Prot database. While 6,953 (12.72%) unigenes were annotated against KO, 8,777 (16.06%) were annotated in KOG. Altogether, BLAST searches against the Nr, Nt, Swiss-Prot, KOG, KO, and GO databases showed that a total of 25,570 (46.80% of 54,628 unigenes) identified unigenes could be annotated with known biological functions.

### 3.3. Gene Ontology Analysis

**Figure 1:**
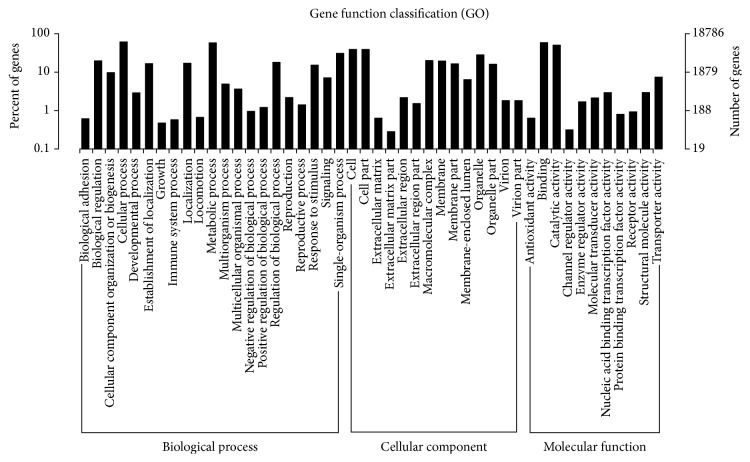
Histogram of gene ontology (GO) classification. The results are summarized in three main categories: biological process, cellular component, and molecular function.

Based on GO annotation, 18,787 unigenes were categorized into 62 subcategories in 3 main categories. As shown in Figure 1, the majority of the unigenes were classified into the molecular functions (16,277, 86.64%), followed by biological processes (14,474, 77.04%) and cellular components (10,345, 55.06%). Under the cellular component ontology, the “cell” and “cell part” subcategories showed a little prevalence, which were similar to the assignment results in leaf transcriptomes [22, 34]. Regarding the molecular function ontology, “binding and catalytic activity” category was obviously larger than the others. The “cellular processes” and “metabolic process” appeared to be low dominant, indicating that positive developmental processes occurred in the leaves and shoots.

### 3.4. KOG Annotation Analysis

As a result of searches of 54,628 unigenes against the KOG database, 8,777 unigenes (16.06%) were ultimately predicted which assigned to 25 functional categories. The low rate of matching partly reflects the fact that* T. sinensis* is a phylogenetically distant species compared with the species included in the eukaryotic KOG database. “General function prediction only” was the largest category with 1,643 unigenes (18.72%), followed by “Posttranslational modification, protein turnover, chaperones” (1,148 unigenes, 13.08%), “Signal transduction mechanisms” (752, 8.57%), “Translation, ribosomal structure and biogenesis” (614, 7.00%), and “Transcription” (537, 6.12%).

### 3.5. KEGG Pathway Analysis

**Figure 2:**
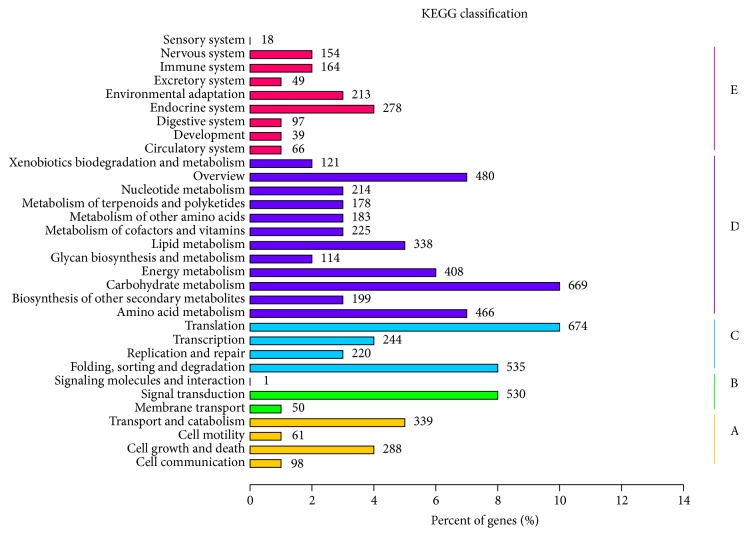
Pathway assignment based on KEGG. (A) Cellular processes; (B) environmental information processing; (C) genetic information processing; (D) metabolism; (E) organismal systems.

KEGG database annotation provided the biochemical pathways and categorizing gene functions [34]. Among 54,628 assembled unigenes, a total of 6,953 unigenes were assigned to 257 KEGG pathways (Figure 2). In the Hierarchy1 Pathway, the highly represented pathways included metabolic pathways (3,595), genetic information processing (1,673), organismal systems (1,078), cellular processes (786), and environmental information processing (581). As shown in Figure 2, the KEGG metabolism category contained 32 subcategories. “Translation,” “Carbohydrate metabolism,” “Folding, sorting and degradation”, and “Signal transduction” appeared to be somewhat dominant in the Hierarchy1 Pathway subcategories. The predicted metabolic pathways were generally involved in carbohydrate, energy, amino acid, lipid and nucleotide, and glycan metabolism, which indicated that the leaves and shoots of* T. sinensis* were undergoing primary material metabolism of multiple materials for growth and development.

### 3.6. Analysis of Differentially Expressed Unigenes

Gene expression was calculated in accordance with the RPKM method (RPKM, unigenes per kilobase per million mapped unigenes), which takes into account the influences of both the sequencing depth and gene length on the read count. Based on the applied criteria [*q* value < 0.005 and log⁡2  (fold change) > 1], 3,710 unigenes (1.19% of all genes) were identified as significant differentially expressed genes (DEGs), which comprised 2087 upregulated genes (accounting for 56.25% of all significant DEGs) and 1623 downregulated genes (accounting for 43.75%) in XC-*L*-4.

Several enriched amino acid biosynthesis pathways were identified through KEGG enrichment analysis. There were 46 DEGs identified among 267 background unigenes that were involved in amino acid biosynthesis pathways. Among those amino acids biosynthesis pathways, in particular, the lysine biosynthesis pathway was identified as being the most enriched one with 7 upregulated DEGs among 10 unigenes. Lysine is an essential amino acid, and an important amino acid for human health, because humans are unable to synthesize it. Here, we chose the lysine pathway for further analysis.

### 3.7. Lysine Biosynthesis Pathway Analysis

**Figure 3:**
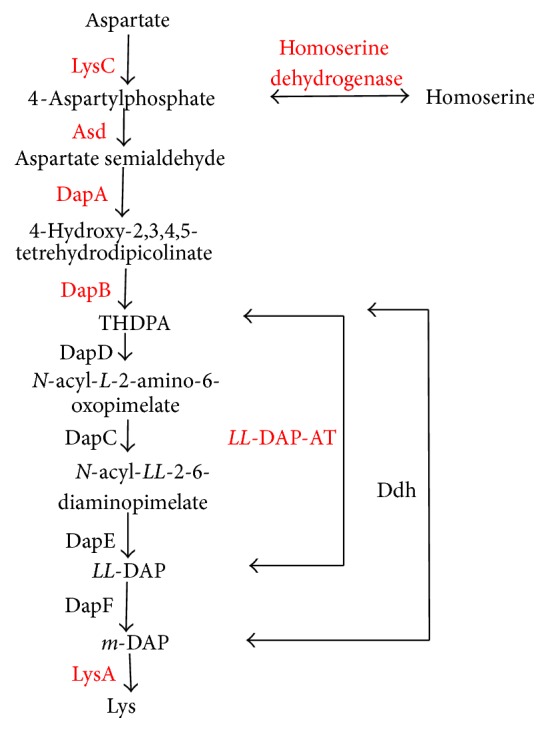
The mechanisms for lysine synthesis. The pathways labeled in the diagram include three variants that use either succinyl-CoA or acetyl-CoA. The first pathways were the acyl-DAP pathways. Another uses Ddh (DAP dehydrogenase) to directly convert THDPA to* m*-DAP.* LL*-DAP-AT directly converts THDPA to* LL*-DAP. Acronyms in the diagram include LsyC, aspartate kinase; Asd, aspartate-semialdehyde dehydrogenase; DapA, dihydrodipicolinate synthase; DapB, dihydrodipicolinate reductase; THDPA,* L*-2,3,4,5-tetrahydrodipicolinate; DapD, THDPA acyltransferase; DapC,* N*-acyl-*L*-2-amino-6-oxopimelate aminotransferase; DapE,* N*-acyl-*LL*-2,6-diaminopimelate deacylase;* LL*-DAP,* LL*-2,5-diaminopimelate; DapF, DAP epimerase;* m*-DAP,* m*-2,6-diaminopimelate; LysA,* m*-DAP decarboxylase;* LL*-DAP-AT,* LL*-diaminopimelate aminotransferase; Ddh,* m*-DAP dehydrogenase. The red words represent upregulated DEGs; the black words represent not DEGs.

The scheme depicts three abbreviated diaminopimelate pathways for the biosynthesis of lysine known in prokaryotes (Figure 3) [35]. All three carry out the same upstream pathway from aspartate to tetrahydrodipicolinate (THDPA) through the sequential action of aspartate kinase (LysC), aspartate-semialdehyde dehydrogenase (Asd), dihydrodipicolinate synthase (DapA) and dihydrodipicolinate reductase (DapB), and the same final reaction from mesodiaminopimelate (*m*-DAP) to lysine catalyzed by* m*-DAP decarboxylase (LysA) [35]. The differences between them lie in the reactions at the center of the pathway from THDPA to* m*-DAP. Lysine biosynthesis in plants is known to utilize the intermediate diaminopimelic acid (DAP). The first type of pathway to have been discovered carried out four sequential reaction steps firstly through a succinyl-CoA-dependent transferase (DapD) or is acetylated by acetyl-CoA-dependent transferase, then DapC (a Glu-dependent type of transamination), and desuccinylase (DapE) to form* LL*-diaminopimelate (*LL*-DAP), and an epimerase (DapF) finally converts* LL*-DAP to* m*-DAP. Another DAP pathway utilizes* m*-DAP dehydrogenase (Ddh) to convert THDPA to* m*-DAP, shortening the central portion of the pathway from four steps to one. A third DAP pathway identified in plant utilizes* LL*-DAP aminotransferase (*LL*-DAP-AT/DapL), which specifically catalyzes the interconversion of tetrahydrodipicolinate and* LL*-diaminopimelate, a reaction requiring three enzymes in the DAP pathway.

**Figure 4:**
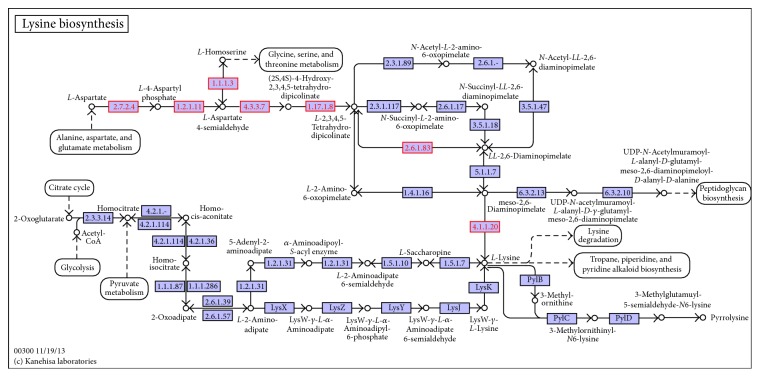
The lysine biosynthesis in* T. sinensis*. Note: The red dots represent upregulated DEGs in XC-*L*-4; the purple dots represent not DEGs.

According to our results, 7 DEGs were identified among ten background unigenes covering all of the genes in the lysine biosynthesis pathways. The 7 unigenes were all upregulated DEGs in XC-*L*-4 (Figure 4, Table S2). Further analyses were followed. comp45867_c0 (2.6879) was annotated as aspartate kinase [LysC, EC:2.7.2.4], which is the first enzyme that catalyzes aspartate into 4-aspartylphosphate. One branch reaction from homoserine to 4-aspartylphosphate was identified in* T. sinensis* as being catalyzed by homoserine dehydrogenase [EC:1.1.1.3], whose transcript level in XC-*L*-4 was higher by 1.827-fold (comp47995_c0). Comp43865_c0 was annotated as aspartate-semialdehyde dehydrogenase [asd, EC:1.2.1.11], responsible for the reaction converting 4-aspartylphosphate to aspartate-semialdehyde, which was upregulated 2.7584-fold in XC-*L*-4 compared with XC-*L*-6. Comp45016_c0 (1.4805) and comp41544_c0 (1.4408) were annotated as DapA [EC:4.3.3.7] and DapB [EC:1.17.1.8], respectively, and found to be upregulated by 1.4805- and 1.4408-fold in XC-*L*-4. We assumed that the THDPA content would be higher in the leaves collected in April than those collected in June.

Among the next three pathways leading from THDPA to* m*-DAP, only the pathway catalyzed by* LL*-diaminopimelate aminotransferase (*LL*-DAP-AT, DapL) [EC:2.6.1.83] showed differences between the two samples collected at two different times; one unigene (comp43351_c0) was identified as DapL, whose transcript level was upregulated 1.8659-fold in XC-*L*-4.* LL*-DAP-AT was first identified in* Arabidopsis* [35] and may be a factor limiting the rate of Lys biosynthesis, potentially showing implications for plant improvement [35]. Therefore, this unigene identified in our database represents a potential key gene for further investigation. The last enzyme, mesodiaminopimelate (*m*-DAP) decarboxylase (LysA), catalyzes the final reaction converting* m*-DAP to lysine. In our transcriptome database, one DEG (comp45712_c0) was identified as LysA [EC:4.1.1.20], which was upregulated by 1.8427-fold.

The above seven unigenes, including LysC, Asd, DapA, DapB, DapL, and LysA, involved in the lysine biosynthesis pathway were all upregulated in the leaves and shoots collected in April.

## 4. Discussion

### 4.1. Characterization of the* T. sinensis* Transcriptome

Transcriptome sequencing has become an important tool because of its low cost and high throughput [20–23]. In this study, we first reported the transcriptome sequencing carried out on* T. sinensis*. 54,628 unigenes were obtained with an N50 value of 1,304 bp and a mean length of 764 bp, which is used for assembly evaluation. Comparable with recently published leaf transcriptomic analyses for other plant species (Table S3), such as* Physalis peruviana* [20],* Quercus pubescens* [21],* Haloxylon ammodendron* [22],* Reaumuria soongorica* [23], and* Salvia splendens* [34], these RNA-Seq data for* T. sinensis* present a fairly high-quality assembly.

The protein homology searches revealed that* T. sinensis* unigenes had higher similarity to* V. vinifera* genes (30.75% of annotated unigenes),* Populus trichocarpa* (24.15%),* Ricinus communis* (23.87%), implying those species protein information would provide valuable reference for further gene discovery, marker development of* T. sinensis*. However, only fewer than half of the unigenes obtained from* T. sinensis* (23,227, 42.51%) exhibited significant matches with those known genome, the vast remaining (31,405, 57.49%) displayed no significant hits. Its insufficient sequences in the Nr database and distinct differences between* T. sinensis* with other species were the part of reasons for the higher proportion of uncharacterized sequences compared with characterized sequences. Therefore, our results afforded a valuable foundation of the gene expression files of* T. sinensis* leaf and shoot for its genetic and genomic research.

### 4.2. Transcriptome Assembly and Gene Annotation

In this study, 21,851 (43.06%) unigenes out of 50,742 identified were successfully annotated using BLAST searches of the public nr, Pfam, Swiss-Prot, GO, COG, and KEGG databases. Through the comprehensive analysis, those distribution patterns annotated in several databases indicated the* T. sinensis* leaves and shootsin the spring were undergoing multiple developmental processes. For example, of the 18,787 sequences annotated with GO terms, 10,361 were assigned to 60 EC (enzymes with catalytic activity) catalogues. Specifically, transferase activity (3557, 34%), hydrolase activity (34%) and oxidoreductase activity (20%) were the most represented enzyme categories. The large number of annotated enzymes within these three groups suggests the presence of genes associated with pathways of primary and secondary metabolite biosynthesis [34].

In early spring,* T. sinensis* was rich in nutrients, including protein (average 3.06 mg/g fresh weight), soluble sugars (3.2 mg/100 g), fats (7.15 mg/100 g), essential amino acids (1.92 mg/g), carbohydrates, and phosphorous [10, 11], vitamin B1, vitamin B2, vitamin C (94 mg/100 g), beta carotene, terpenes and flavonoids [36, 37]. The predicted results make it possible that were general consistent with the contents of the various compounds reported in the leaves and shoots of* T. sinensis*. Therefore, the obtained transcriptomes from* T. sinensis* will provide genetic information on the biosynthetic routes of primary and secondary metabolism.

However, we should notice more than half of unigenes generated were not annotated according to existing databases. Therefore, according to these results, there is a need to generate a large collection of unigenes and further characterize the gene structures and expression patterns in* T. sinensis*.

### 4.3. Analysis of Differentially Expressed Unigenes in Lysine Biosynthesis

According to our results, the lysine biosynthesis pathways was identified as being the most enriched one because of 7 DEGs among ten background unigenes. The seven unigenes, including LysC, Asd, DapA, DapB, DapL and LysA, were all upregulated in the leaves and shoots collected in April. The obtained results showed that the leaves collected in April were rich in several essential amino acids, especially lysine, which showed significantly higher levels, consistent with the results of nutrient analysis [10, 38]. Animals cannot produce lysine, and they therefore must rely on a dietary source, which is derived primarily from crop plants. Because some crops do not accumulate sufficient lysine to allow them to be used as complete nutritional sources, there has been significant interest in improving nutritional quality by enhancing lysine content [39]. Thus, if* T. sinensis* can accumulate high levels of lysine, it will become an interesting crop, both for research on lysine synthesis and for quality improvement. It is known that, in plants, the control of lysine homeostasis is complex and plays a role as significant as that of biosynthesis [13, 40]. In the description of our transcriptome, most of the catabolic genes associated with plant lysine synthesis were identified, demonstrating our understanding of the exact pathway present in plants [35]. Recent genetic, molecular, and biochemical evidence suggests that lysine synthesis and catabolism are regulated by novel concerted mechanisms [38]. The recent advances in our understanding of the regulation of lysine metabolism in plants may also prove valuable information for the future production of high-lysine crops.

## 5. Conclusions

We performed a* de novo* transcriptome sequencing analysis of* T. sinensis* leaf tissues using the Illumina platform. To the best of our knowledge, this is the first report on the whole transcriptome of* T. sinensis*. More than 4.2 Gb of data were generated and assembled into 54,628 unigenes. A total of 25,570 could be annotated with known biological functions. 12,097 unigenes were assigned to 5 main categories, including 123 KEGG pathways. Based on similarities to known proteins, proteins involved in primary metabolite biosynthesis were identified, including proteins related to carbohydrate, amino acid, energy, and lipid biosynthesis. Analysis of differentially expressed unigenes between the two libraries showed that the lysine biosynthesis was an enriched KEGG pathway, and candidate genes involved in the lysine biosynthesis pathway in* T. sinensis* leaves and shoots were identified. This research is essential and useful for understanding the transcriptome characteristics of* T. sinensis*. The results obtained from this study will also serve as a useful genomic dataset to accelerate research on metabolic mechanisms and functional genomics in* T. sinensis*.

## Supplementary Material

Supplementary Table S1: Assessment of assembly quality for *Toona sinensis* Roem libraries of two different genotypes.

## Figures and Tables

**Table 1:** Summary of *de novo* sequence assembly for *Toona sinensis*.

Assembly parameters	Transcripts	Unigenes
Transcripts generated	125,884	54,628
200–500 bp	45596	34113
500–1 kb	21690	8219
1 kb-2 kb	30384	7138
>2 kb	28214	5158
N50 value (bp)	2026	1505
Minimum length (bp)	201	201
Mean length (bp)	1290	764
Median length (bp)	880	364
Maximum length (bp)	18,462	18,462
N50 (bp)	2133	1505
N90 (bp)	579	277
Total nucleotides (bp)	162,340,052	41,755,962
