# Supplementary material for: De Novo Assembly and Comparative Transcriptome Analysis Provide Insight into Lysine Biosynthesis in Toona sinensis Roem
Source: Int J Genomics. 2016 Jun 7;2016:6735209. doi: 10.1155/2016/6735209 (PMC4914729; doi:10.1155/2016/6735209)
Supplement: Supplementary file 1 — Supplementary Table S1: Assessment of assembly quality for Toona sinensis Roem libraries of two different genotypes. [file 6735209.f1.pdf]

Additional file

Table S1 Assessment of assembly quality for two *T. sinensis* libraries

Sample	Raw Reads	Clean Reads	Clean Bases	Error(%)	Q20(%)	Q30(%)	GC Content(%)
XC_L_4_1	22841245	21306359	2.13G	0.04	96.92	90.41	45.09
XC_L_4_2	22841245	21306359	2.13G	0.03	97.47	91.99	45.09
XC_L_6_1	30874262	29687215	2.97G	0.03	97.23	91.35	43.44
XC_L_6_2	30874262	29687215	2.97G	0.03	97.62	92.55	43.46

Table S2. RPKM values and comparison of DEGs in Lysine biosynthesis pathway under the condition of XC_L_4 vs XC_L_6

Gene ID	RPKM value (XC_L_4)	RPKM value (XC_L_6)	$Log2^{Fold\ change}$	KO Name
comp45867_c0	42.6664	6.6216	2.6879	Aspartate kinase (LysC) [EC:2.7.2.4]
comp47995_c0	50.9807	14.3693	1.827	Homoserine dehydrogenase [EC:1.1.1.3]
comp43865_c0	50.5255	7.4672	2.7584	Aspartate-semialdehyde dehydrogenase (Asd)[EC:1.2.1.11]
comp45016_c0	98.7750	35.3986	1.4805	4-hydroxy-tetrahydrodipicolinate synthase (DapA) [EC:4.3.3.7]
comp41544_c0	108.9146	40.1190	1.4408	4-hydroxy-tetrahydrodipicolinate reductase (DapB) [EC:1.17.1.8]
comp43351_c0	84.9979	23.3191	1.8659	LL-diaminopimelate aminotransferase (ll-DAP-AT/DapL), [EC:2.6.1.83]
comp45712_c0	74.5592	20.7871	1.8427	Diaminopimelate decarboxylase(LysA) [EC:4.1.1.20]

Table S3. Comparative N50 and average length with other plant species recently published in transcriptome data

Species	N50 (bp)	Average length (bp)	Reference
Toona sinensis	1,304	764	In the paper
Reaumuria soongorica	1,109	677	Shi et al., 2013[23]
Haloxylon ammodendron	1,345	728	Long et al., 2014[22]
Salvia splendens	1,304	772	Ge et al., 2014[33]
Physalis peruviana	1,438	743	Garzón-Martínez et al, 2012[20]
Quercus pubescens	910	618	Torre et al., 2014[21]
